# Bismuth Telluride nanocrystal: broadband nonlinear response and its application in ultrafast photonics

**DOI:** 10.1038/s41598-018-20559-y

**Published:** 2018-02-05

**Authors:** Guobao Jiang, Jun Yi, Lili Miao, Pinghua Tang, Huihui Huang, Chujun Zhao, Shuangchun Wen

**Affiliations:** 1 Key Laboratory for Micro-/Nano- Optoelectronic Devices of Ministry of Education, School of Physics and Electronics, Hunan University, Changsha, 410082 China; 2 Hunan Key Laboratory for Micro-Nano Energy Materials and Devices, Xiangtan University, Xiangtan, 411105 China

## Abstract

We come up with a hybrid liquid exfoliation method to prepare bismuth telluride nanocrystals efficiently and cost-effectively. The nonlinear transmittance of the nanocrystals has been characterized with Z-scan technique, which can manifest its broadband saturable absorption behavior experimentally. The as-fabricated nanocrystals were integrated onto fiber end facet to form a fiber compatible nonlinear absorption device with optical deposition method, which was then used to modulate the fiber laser with different cavity configurations to deliver pulsed laser successfully. The noise-like pulse and dissipative soliton have been obtained with wavelength centered at 1562 nm and 1068 nm, respectively. These results confirm the effectiveness of the hybrid liquid exfoliation method to prepare bismuth telluride into nanocrystals, and the broadband nonlinear optical response and ultrafast photonics application potential of the nanocrystals.

## Introduction

Ultrafast fiber lasers are fundamental building blocks of many photonics systems used in optical communication, industrial and medical applications as well as for scientific researches for its excellent beam quality, compact structure, high efficiency, etc^1^. The ultrafast fiber laser can be realized by passively mode-locking method with saturable absorber (SA), whose light transmittance coefficient increases with the increasing of light intensity. The widely-used commercial SA is the semiconductor saturable absorber mirrors (SESAMs)^2,3^. However, the SESAMs are expensive for fabrication and have limited operation bandwidth^2^. With the evolving technologies to find excellent broadband and cost-effective SAs, the SA with different dimensions have been developed and validated in ultrafast photonics, such as quantum dots in zero-dimension^4^, carbon nanotube in one-dimension^5^, and graphene^6^ and graphene-like materials in two-dimensional extensions^7–10^. Driven by the requirements to obtain highly stable, ultrashort, high power ultrafast pulsed fiber laser, the excellent SAs with broadband nonlinear response, low cost and controllable modulation depth need to be explored and developed by controlling and manipulating the dimension and size.

Topological insulator (TI) is extensively studied in recent years as a new state of quantum matter, which possesses insulating gapped bulk state and topologically protected metallic gapped surface state^11^. With the special singular quantum-mechanical properties, TI has shown great application potentials in thermoelectricity, electronics and photonics^12–17^. Derived from the Dirac-like linear band surface state, TI can be developed into an ultra-broadband nonlinear optical material^18^, which is similar with graphene^19^. Since the first reported mode-locked fiber laser based on TIs (Bi_2_Se_3_, Bi_2_Te_3_)^20,21^, a large number of research works focused on pulsed lasers with wavelength ranging from 1 μm to 3 μm based on TI-SA^22–32^. As for the typical TI, bismuth telluride Bi_2_Te_3_ bulk material and nanosheets have been investigated^23–25,27–29,31,32^. With the reduced size, especially at least one dimension down to less than 100 nm, the Bi_2_Te_3_ nanomaterial, i.e. Bi_2_Te_3_ nanocrystal, has been used as an efficient thermoelectric material for cooling and power generation applications^33,34^. The electron confinement in nanocrystal provides powerful means to manipulate the electronic, optical, and magnetic properties of a solid material. However, the nonlinear optical response of the nanocrystal has not been fully explored yet. In addition, many complicated methods have been used to produce high quality low-dimensional Bi_2_Te_3_, such as reactions from the atomic or molecular scale synthesis^35,36^, mechanical or chemical exfoliation^33,37^. However, these methods suffered from the complicated fabrication process and low yield. Therefore, cost effective and high efficient preparation method of Bi_2_Te_3_ in nanoscale is still an important issue.

Besides the SA, the intra-cavity dispersion and nonlinear management have important impact on the high-performance output of mode-locked fiber laser. By changing the parameters of fiber laser cavity (pump power, polarization state, net dispersion, etc), there are various interesting solitons can be obtained in mode-locked fiber laser, such as noise-like pulse^38,39^, dissipative soliton^40,41^, bound state soliton^42^, vector soliton^43,44^ and so on. The noise-like pulse, as a typical mode-locked output in fiber laser, has attracted a great deal of attention in recent years for it can offer a platform to study the fundamental physical theory for pulse evolution in nonlinear fiber-optic media, such as rogue wave, which is a counterpart of the infamous water waves in the sea^45–48^. Noise-like pulse has also shown its potentials in supercontinuum generation and micromachining on capable of producing large energy pulses^49,50^. As a counterpart, the dissipative soliton also has its unique advantages in large energy pulse and high peak power regime. The operation of dissipative soliton in a large normal dispersion laser cavity could generate large energy and strongly chirped pulses^51,52^, which could be amplified and then compressed into ultrashort high peak power pulses.

In this work, we have developed a high efficient liquid exfoliation method to fabricate Bi_2_Te_3_ nanocrystals. Then the exfoliated Bi_2_Te_3_ nanocrystals were centrifuged to separate the centrifugate and nanocrystals supernatant. The fabricated Bi_2_Te_3_ nanocrystals supernatant were spin coated onto optical quartz substrate and the saturation absorption of Bi_2_Te_3_ nanocrystals was verified under high peak power laser illumination with Z-scan technique. When Bi_2_Te_3_ nanocrystals was introduced into the fiber laser resonators as SA, the broadband mode-locked outputs in different pulsation regimes were realized.

## Experimental Results

### Characterizations of Bi_2_Te_3_ nanocrystals

**Figure 1:**
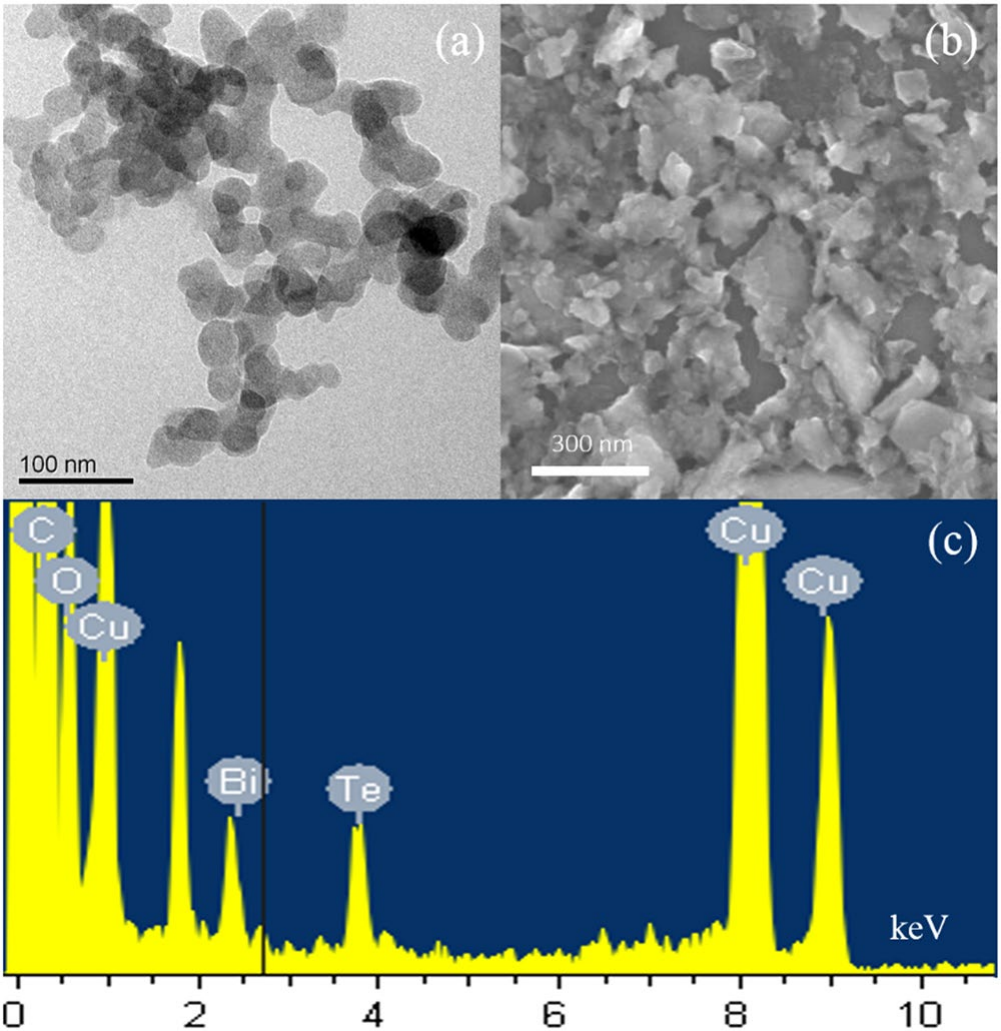
The HRTEM image of the exfoliated Bi_2_Te_3_ nanocrystals. (**a**) SEM image of the exfoliated Bi_2_Te_3_ nanocrystals. (**b**) HRTEM image of the layered structure Bi_2_Te_3_ nanocrystals. (**c**) EDS analysis of the Bi_2_Te_3_ nanocrystals.

Bi_2_Te_3_ nanocrystals have been prepared by the mixed solvents liquid phase exfoliation method, which is a simple and effective technique to prepare two dimensional (2D) materials that are difficult to exfoliate from layered bulk crystals towards several layered structures. To investigate the exfoliation result, the morphology and size of the as-prepared Bi_2_Te_3_ sample was characterized with high resolution transmission electron microscope (HRTEM) and scanning electron microscope (SEM). Figure 1(a) shows the TEM image of the prepared Bi_2_Te_3_ nanocrystals. Almost all of the prepared Bi_2_Te_3_ nanocrystals were observed to be less than 50 nm wide. The SEM image in Fig. 1(b) tells us that the Bi_2_Te_3_ powders are well exfoliated. The energy dispersive spectrum (EDS) shows that the examined sample contains not only Bi_2_Te_3_, but also C, O and Cu elements. The C, O elements are introduced by the 1-Methyl-2-pyrrolidinone (NMP), N-Octyl pyrrolidone (N8P) and the lacey support films. The Cu elements was the substrate of the lacey support films.

### Nonlinear optical responses

In order to investigate the broadband nonlinear property of the exfoliated Bi_2_Te_3_ nanocrystals, we used open-aperture Z-scan system to measure the nonlinear absorption at different wavelength. The Bi_2_Te_3_ sample moved along the optical axis around beam focus to get the dependence transmittance about the input intensity.

**Figure 2:**
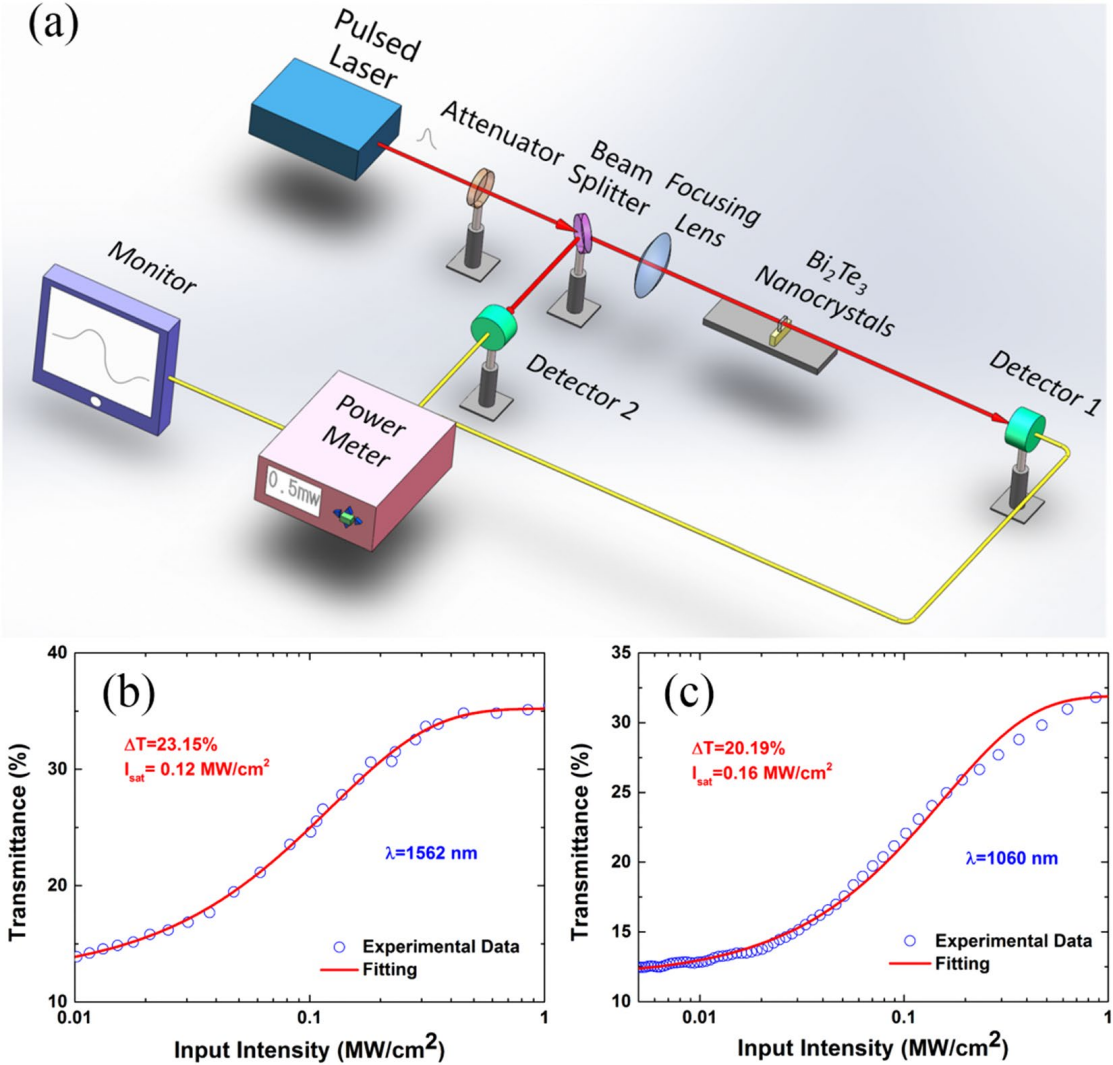
Characterization of the saturable absorption property of the Bi_2_Te_3_ nanocrystals. (**a**) Z-scan measurement system. (**b**) The open-aperture measurement of the Bi_2_Te_3_ nanocrystals at 1562 nm. (**c**) The open-aperture measurement of the Bi_2_Te_3_ nanocrystals at 1060 nm.

The transmittance with input power intensity was fitted with the formula:

1 $$T(I)=1-{\rm{\Delta }}\alpha \ast exp(-I/{I}_{sat})-{\alpha }_{ns}$$

where, *T(I)* is transmittance, *Δα* is modulation depth, *I* is input intensity, *I*_sat_ is saturation intensity, and *α*_ns_ is non-saturable absorbance. The fitted results were shown in Fig. 2, from which the conclusion can be easily achieved that the transmittance increases with the increasing light intensity. From the best fit in Fig. 2(b), the modulation depth and saturable intensity are extracted to be 23.15% and 0.12 MW/cm^2^ at 1562 nm, respectively. Figure 2(c) showed the nonlinear transmittance of Bi_2_Te_3_ nanocrystals with modulation depth and saturable intensity of 20.19% and 0.16 MW/cm^2^ at 1060 nm, respectively. The Bi_2_Te_3_ nanocrystals shows different nonlinear optical response, which could be attributed to the different wavelength and the available laser source adopted with different operating parameters. These results suggest that Bi_2_Te_3_ nanocrystals possess broadband saturable absorption and have application potentials in broadband ultrafast photonics.

### Mode locked fiber lasers with Bi_2_Te_3_ nanocrystals

We designed two fiber laser cavities schematically using Erbium and Ytterbium doped fiber as active fiber in order to evaluate the broadband mode-locking ability of Bi_2_Te_3_ nanocrystals.

**Figure 3:**
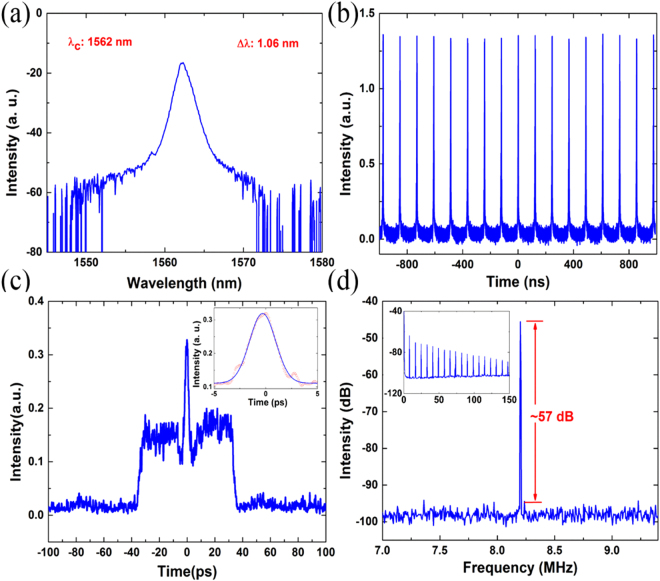
The characteristics of the noise-like pulse output. (**a**) Output spectrum. (**b**) The pulse train. (**c**) The autocorrelation trace, inset: the pulse width of the central peak. (**d**) Radio frequency spectrum, inset: wide span radio frequency spectrum.

For the Er-doped fiber laser mode-locked by Bi_2_Te_3_ nanocrystals, a broadened smooth optical spectrum was captured with the spectrum analyzer, while pump power was tuned exceeded 270 mW and polarization controllers (PCs) slightly adjusted. We could observe a clear distinction of the output spectrum in comparison with that of the fundamental soliton regime, without explicitly Kelly sidebands in Fig. 3(a). A series stable pulses were detected with the oscilloscope and showed a fundamental frequency of the cavity, as can be seen in Fig. 3(b). Apparently, the fiber laser operated in a mode-locked regime. Considering the differences between the spectrum and general mode-locked soliton characteristics, we further measured the auto-correlation trace. The auto-correlation trace of the output pulses was shown in Fig. 3(c), which shows a typical noise-like pulse autocorrelation profile that a coherent spike riding on a pulse envelope. With the envelop extending to about 80 ps, the width of the coherent spike was estimated to be 3.04 ps which indicates that these pulses have an averaged pulse width of about 1.97 ps. One point should be noted that the width of the pulse envelope was too broad to be measured precisely with our available autocorrelator whose accurate measurement maximum pulse width is ~20 ps. Taken together, these results confirmed the noise-like pulse output. The radio frequency spectrum, as shown in Fig. 3(d), illustrated the signal to noise ratio of the pulse train can be over 57 dB. The fundamental repetition rate was measured to be 8.20 MHz, which matched well with the laser cavity length. The experimental results meant a stable noise-like pulse mode locking operation.

**Figure 4:**
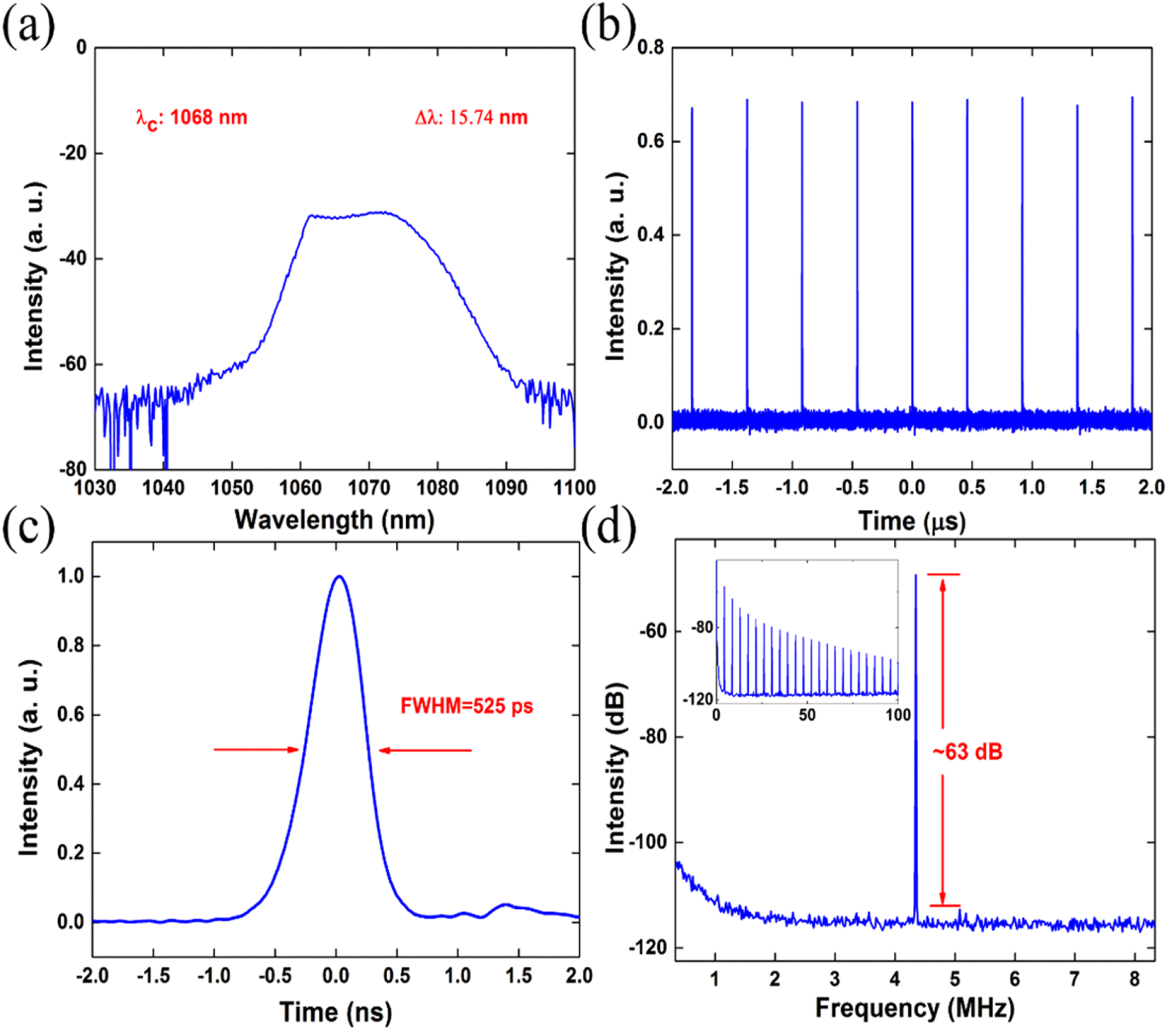
The characteristics of the dissipative soliton output. (**a**) The spectrum. (**b**) The pulse train. (**c**) Single pulse profile. (**d**) Radio frequency spectrum, the inset: wide span radio frequency spectrum.

Self-started mode-locking operation was also obtained in the Yb-doped fiber laser. The output characterizations were shown in Fig. 4 with a pump power of 240 mW. Typical dissipative soliton spectrum with a center wavelength at 1068 nm and 15.74 nm 3 dB bandwidth was shown in Fig. 4(a). A series of stable pulse train in Fig. 4(b) confirmed the mode-locking operation. The pulse train, measured by the oscilloscope, showed a pulse to pulse interval of 230 ns, which matched well with the cavity round trip. The oscilloscope can also characterize the Gaussian pulse profile with a width of 525 ps in Fig. 4(c). Figure 4(d) showed the radio frequency spectrum of 4.35 MHz, which corresponded to the cavity repetition rate well. The signal to noise ratio was 63 dB without additional frequency peak.

## Discussions

To examine the broadband nonlinear optical response of the exfoliated Bi_2_Te_3_ nanocrystals, broadband Z-scan measurements are performed. The saturable absorption curves show that TI nanocrystals exhibit broadband saturable absorption. The as fabricated Bi_2_Te_3_ nanocrystals were successfully applied in the fiber lasers to obtain the mode-locking, which fully affirmed the broadband saturable absorption property of Bi_2_Te_3_ nanocrystals.

In the mode-locked Er-doped fiber laser, the noise-like pulse was observed in the mode-locked Er-doped fiber laser cavity. As for the mode-locked fiber laser in abnormal dispersion regime, the mode-locked pulses would split into multiple pulses easily for the peak power clamping effect of the laser cavity. In our experiment, 6 m length active fiber could offer a large gain and produce a large intracavity energy. The formation of noise-like pulses is caused by the combined effect of soliton collapse and positive cavity feedback^53^. The initial soliton will break into multiple pulses by the peak power clamping effect. The newly formed pulse was amplified and split too. Repeatedly, the pulse will split into a pulse cluster, in which the pulses were too close to tell apart in time domain^54,55^. The other consideration was the long gain fiber with a normal dispersion, which could compress the pulse width in time domain^56^. All these pulses bunched together and formed a pulse packet “noise-like pulse”.

We observed dissipative solitons in mode-locked Yb-doped fiber laser like other literatures^43,57^. The formation of the dissipative soliton should be attributed to the large normal group velocity dispersion (GVD) of fibers at 1 μm. Dissipative solitons derived from a normal dispersion fiber laser cavity with mutual effects of gain, loss, nonlinear Kerr effect, net normal dispersion and intracavity gain bandwidth filtering^58^. Due to the normal dispersion, dissipative solitons are strongly frequency chirped. Taking advantage of this feature, we could produce high peak power with dissipative solitons amplified and compressed^51^.

Considering the broadband spectrum of noise-like pulse and dissipative soliton at different wavelength, the broadband nonlinear optical response of Bi_2_Te_3_ nanocrystals are fully confirmed.

## Conclusions

To this end, we showed a high efficient hybrid liquid exfoliation method to prepare Bi_2_Te_3_ nanocrystals. We propose that this hybrid liquid exfoliation method can be employed, as it also works in exfoliating other layered materials. As such, we expect to extend it to graphene, transition metal dichalcogenides, black phosphorene and other layered compounds. Moreover, we performed nonlinear optical characterization of the exfoliated products, and validated the broadband nonlinear response of the Bi_2_Te_3_ nanocrystals. By optical deposition method, the prepared Bi_2_Te_3_ nanocrystals were deposited onto fiber end facet to form an SA and then introduced into ring fiber laser cavities. As a result, passively mode-locking operations were achieved at both 1562 nm and 1068 nm with noise-like pulse and dissipative soliton, respectively. This study offers a cost-effective strategy to produce high quality Bi_2_Te_3_ nanocrystals, and also validates the nanocrystals’ broadband nonlinear optical response and effectiveness in ultrafast photonics applications.

## Methods

### Bismuth telluride nanocrystals preparation

Here, Bi_2_Te_3_ nanocrystals were fabricated with a simple top-down method. Bulked Bi_2_Te_3_ (20 mg) was grinded into powder and then transferred into a centrifuge tube with 10 mL hybrid dispersant (10% NMP and 90% N8P percentage in volume). Then the hybrid liquid was put in a water bath ultrasonicator for ultrasonic processing for 2 hours. The as produced mixed liquor was centrifuged for 20 minutes at 7000 rpm to remove the unexfoliated Bi_2_Te_3_. The supernatant products-Bi_2_Te_3_ nanocrystals’ dispersion were what we want.

### Experimental setups of open-aperture Z-scan measurement

In order to investigate the broadband nonlinear property of the exfoliated Bi_2_Te_3_ nanocrystals, we performed an open-aperture Z-scan system to characterize the nonlinear absorption at different wavelength of Bi_2_Te_3_ nanocrystals. The measurement system was shown in Fig. 2. A mode-locked erbium doped fiber laser (Center wavelength: 1562 nm, Pulse duration: 1.5 ps, Repetition rate: 20.8 MHz) and a mode-locked ytterbium doped fiber laser (Center wavelength: 1060 nm, Pulse duration: 130 ps, Repetition rate: 2.7 MHz) were used as laser sources. The incident light was divided into two equal part with a beam splitter: one was used as a reference beam detected by a photodetector, and the other part was focused perpendicularly to the Bi_2_Te_3_ sample. The sample placed on a linear motorized translation stage, which was controlled by a computer. The intensity of the laser passing through Bi_2_Te_3_ sample was monitored with another photodetector. By adjusting the distance between the sample and the focus of the lens, the light intensity can be tuned in large scale. The transmittance was calculated by the output power dividing the reference power.

### Bismuth telluride nanocrystals-based SA fabrication

**Figure 5:**
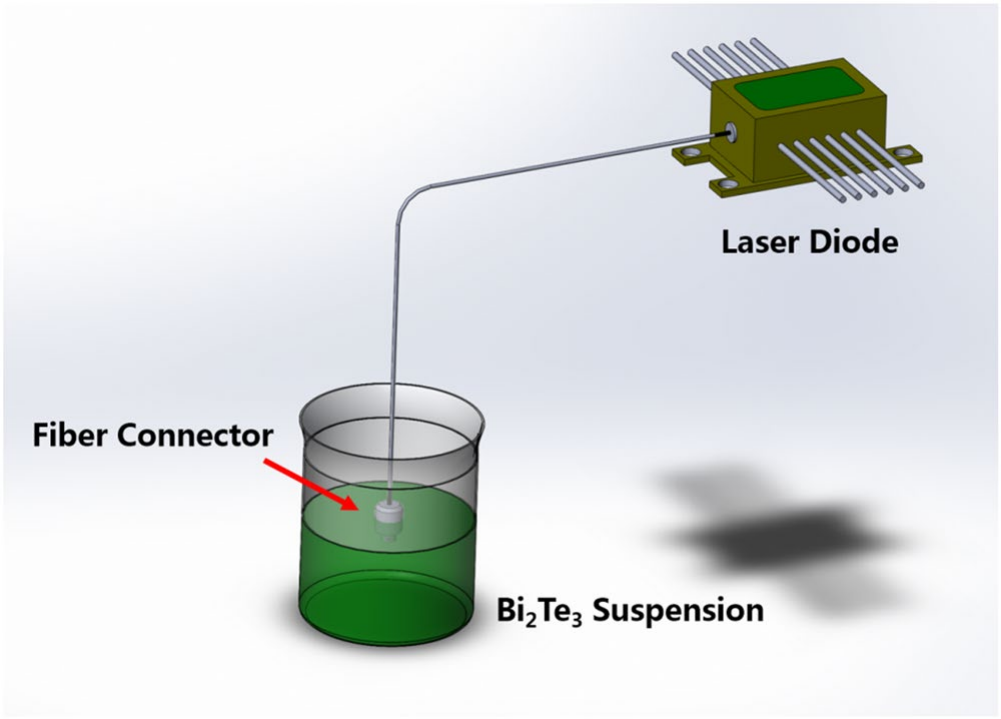
Schematic diagram of optical deposition setup.

The prepared Bi_2_Te_3_ nanocrystals was deposited onto the fiber end with optical deposition method, as shown in Fig. 5. A fiber connector was directly spliced to the laser diode, and the fiber end facet was immersed in Bi_2_Te_3_ dispersion. After turning on the laser diode, the light was injected into the solution through the fiber connector. The Bi_2_Te_3_ nanocrystals was deposited onto the fiber end under the light illumination. This was owing to two possible physical mechanics: optical trapping and heat convention effects^59^. Corresponding to these factors, the deposition efficiency depends on several parameters, such as incident light power, illumination time and concentration of the Bi_2_Te_3_ suspension. We set the laser diode power 40 mW and illumination time 5 minutes. The concentration of Bi_2_Te_3_ suspension was the original fluid concentration produced by hybrid liquid exfoliation.

### Bismuth telluride nanocrystals as passively mode-locker in fiber lasers

**Figure 6:**
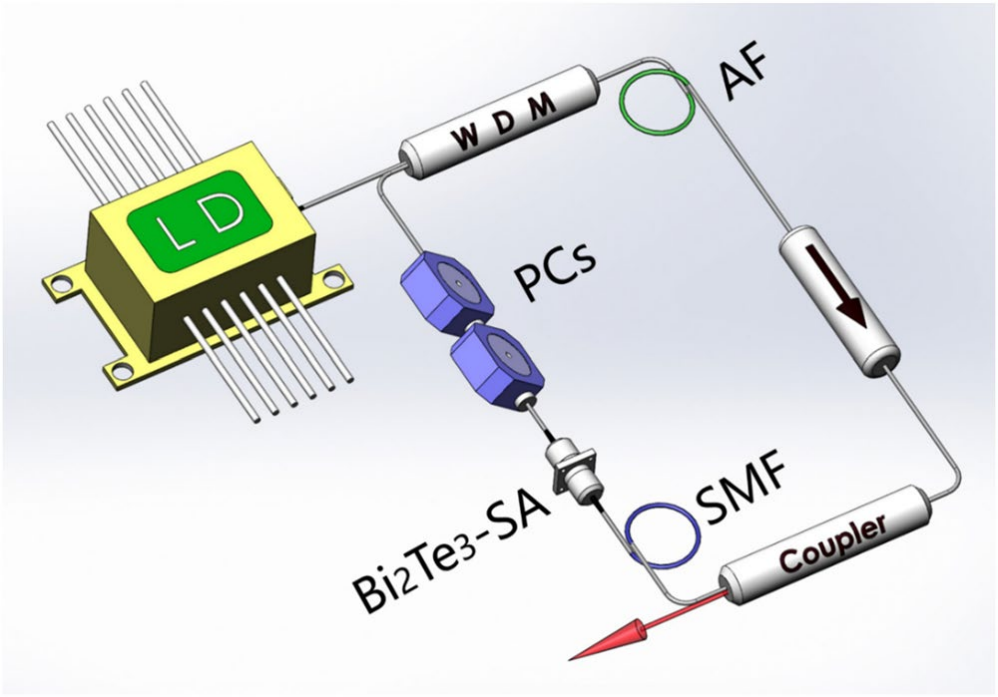
Experimental setup of the Bi_2_Te_3_-SA based fiber laser. LD: laser diode. WDM: wavelength division multiplexer. AF: active fiber. ISO: isolator. PCs: polarization controllers.

The schematic setup of the passively mode-locked fiber lasers based on Bi_2_Te_3_ was shown in Fig. 6. In the erbium doped fiber laser, a 6 meters length Er-doped fiber (EDF) with a normal GVD of 35 ps^2^/km was used as active fiber (AF), which was pumped by a 975 nm laser diode. A 980/1550 nm wavelength division multiplexer (WDM) was set between the AF and laser diode to introduce the pump light into the AF and to collect the feedback laser. A polarization independent isolator (ISO) was used to force the unidirectional light propagation after the AF. A 10% output coupler was used to monitor the mode-locked pulses. A piece of 10 m single mode fiber (SMF: SMF-28) with a GVD of −23 ps^2^/km was used to fix the GVD at abnormal region. Polarization controllers were inserted to modify the mode-locked pulse shape between the SA and WDM. The total cavity length was measured to be 24.4 m, including 6 m length EDF, 10 m length SMF (SMF-28), and 8.4 m length SMF (SMF-28) optical pigtail. The total dispersion of the EDF laser was −0.213 ps^2^. The output mode-locked pulses performance was monitored with an optical spectrum analyzer (Ando AQ-6317B) and a real-time oscilloscope with a bandwidth of 4 GHz (Agilent DSO9404A) combined with a 5 GHz photodetector (Thorlabs SIR5).

Within the Ytterbium doped ring fiber laser, a piece of 0.65 m Yb-doped fiber (LIEKKI Yb1200-4/125) with a GVD 24 ps^2^/km was used as AF. The 980 nm laser diode was introduced as a pump using a 980/1064 nm WDM. The coupler, SMF (HI 1060), Bi_2_Te_3_ nanocrystals SA and PCs were spliced into the cavity at the same position as in the Er-doped fiber laser. A 1% output coupler was used to maximize the pulse energy in the cavity. The total cavity length was about 45.98 m, including a 40 m length SMF (HI 1060) with a GVD of 22 ps^2^/km, which was used to optimize the mode-locking result. The total dispersion of the YDF laser was 0.982 ps^2^. The output pulses were monitored with the same measuring system as mentioned above.
